# Effects of β‐alanine supplementation on physical performance, cognition, endocrine function, and inflammation during a 24 h simulated military operation

**DOI:** 10.14814/phy2.13938

**Published:** 2018-12-18

**Authors:** Alyssa N. Varanoske, Adam J. Wells, Gregory J. Kozlowski, Yftach Gepner, Cheyanne L. Frosti, David Boffey, Nicholas A. Coker, Idan Harat, Jay R. Hoffman

**Affiliations:** ^1^ Institute of Exercise Physiology and Wellness, Educational and Human Sciences, Sport and Exercise Science University of Central Florida Orlando Florida

**Keywords:** Carnosine, ergogenic aid, intracellular buffering capacity, sleep deprivation, soldiers

## Abstract

Sustained military operations (SUSOPs) are associated with performance decrements and cognitive dysfunction. β‐Alanine (BA) supplementation may have a role in increasing soldier resiliency by enhancing muscle‐buffering capacity and reducing oxidative stress. The purpose of this study was to examine the effects of BA on physical performance, cognition, endocrine function, and inflammation during a 24 h simulated SUSOP. Nineteen males were randomized into one of two groups: BA (*n *=* *10) or placebo (*n *=* *9; PLA) (12 g/day) for 14 days preceding the 24 h SUSOP. Assessments were performed at 0 h (0H), 12 h (12H), and 24 h (24H) during the SUSOP. No changes in visual tracking ability, jump power, or upper‐body muscular endurance were observed between groups or time points (*P*'s* > *0.05). Increases in subjective feelings of soreness and fatigue were noted at 12H compared to 0H (*P < *0.05) in PLA, but not in BA. Visual reaction time for PLA was slower at 24H compared to 0H (*P *=* *0.035), and PLA made more errors on reaction time testing at 12H compared to BA (*P *=* *0.048), but motor reaction time was faster (*P *=* *0.016) for PLA. Simulated litter carry and 1 km run completion times increased at 24H compared to 0H in both groups (*P < *0.05), however, PLA had a longer 1 km time compared to BA at 24H (*P *=* *0.050). Increases in inflammatory and endocrine markers were observed over the SUSOP, with no differences between groups. BA supplementation appears to maintain some aspects of cognition and physical performance during a 24 h SUSOP, with no effects on endocrine function or inflammation.

## Introduction

Sustained military operations (SUSOPs) and simulated operational training are often accompanied by periods of prolonged wakefulness, partial sleep loss, or complete sleep deprivation. SUSOPs usually require extreme physical exertion, limited feedings, and psychological stress, exacerbating the overall physiological stress placed on the body (Haslam 1984; Nindl et al. 2002; Lieberman et al. 2006). During periods of prolonged operational stress, severe impairments in cognitive function and physical performance have been reported (Nindl et al. 2002; Lieberman et al. 2006). Nindl et al. (2002), examining the effects of a 72 h SUSOP where soldiers endured prolonged physical work (4500 kcal/day), sleep restriction (~2 h per day), and undernutrition (1600 kcal/day) reported significant decreases in fat‐free mass, fat mass, squat jump power, total work, box‐lift performance, and wall‐building performance. Additionally, mood states tended to become more negative throughout the 72 h period. Similarly, Lieberman et al. (2006) observed significant decrements in vigilance, reaction time, and short‐term memory, along with significantly decreased mood states following 4 days of sustained operations. Interestingly, Lieberman et al. (2006) also observed that cognitive function appeared to decline to a greater extent than physical performance. For soldiers, the decrease in physical and cognitive abilities can increase the risk of accidents and errors, which may ultimately lead to a failed mission (Goh et al. 2001). It has been estimated that ~80–85% of military accidents are a result of diminished cognitive function (Thomas and Russo 2007).

In addition to having detrimental effects on physical performance and cognitive function, SUSOPs and sleep deprivation can also increase the production of reactive oxygen species, increasing oxidative stress and cellular damage (Boonstra et al. 2007; Kalinchuk et al. 2010). Oxidative damage within the brain has also been shown to be one of the factors associated with symptoms of post‐traumatic stress disorder (Wilson et al. 2013). Sleep deprivation has been reported to affect the immune system through increased production of inflammatory cytokines such as interleukin‐6 (IL‐6), tumor necrosis factor‐alpha, and C‐reactive protein (CRP) as well as elevating nonspecific defense immune mechanisms (Dinges et al. 1994; Shearer et al. 2001). Sleep deprivation also decreases muscle glycogen replenishment, inhibiting recovery following exercise (Kong et al. 2002). Adequate sleep can help restore immune and metabolic functioning by facilitating recovery and reducing inflammation.

To combat the decline in physical and cognitive performance associated with SUSOPs, the use of dietary supplements may be an effective intervention. In the past several years, a number of studies examining β‐alanine (BA) supplementation have been shown to have promising effects on several aspects of military performance (Hoffman et al. 2014, 2015a). These studies reported that 4 weeks of BA supplementation (6 g/day) in soldiers performing intense military training was more effective in maintaining lower body power, peak jump power, 50 m casualty carry, cognitive function, target engagement speed, and shooting accuracy compared to soldiers consuming a placebo (PLA). Subsequent research using animal models demonstrated that 30 days of BA feedings may increase resiliency to post‐traumatic stress disorder and mild traumatic brain injury in animals exposed to a predatory scent stress (Hoffman et al. 2015b) and a low‐pressure blast wave (Hoffman et al. 2017) respectively. The efficacy and resiliency demonstrated in these studies were attributed to elevations in both muscle and brain carnosine (β‐alanyl‐L‐histidine). Carnosine acts as an intramuscular buffer, especially during highly‐intense activity (Harris et al. 2006). The importance of BA supplementation is that it is the rate‐limiting precursor to carnosine formation (Harris et al. 2006). Besides acting as an intramuscular buffer, carnosine has been shown to have various other physiological roles in the body, including acting as an antioxidant, antiglycating, and ion‐chelating agent (Kohen et al. 1988; Boldyrev et al. 2013). Elevations in carnosine content within the brain may serve as a neural protectant through its action as an antioxidant (Kohen et al. 1988), potentially reducing neurodegeneration associated with the inflammatory response to stress, leading to improved cognitive function and mood states (Thomas and Russo 2007; Murakami and Furuse 2010; Hoffman et al. 2015a, 2017). BA supplementation has been previously demonstrated to increase brain carnosine concentrations in murine models (Murakami and Furuse 2010; Hoffman et al. 2015b). However, similar results have not been demonstrated in humans (Hoffman et al. 2015a; Solis et al. 2015). Elevations in brain carnosine also appear to increase the expression of brain‐derived neurotropic factor (BDNF) in the hippocampus and cerebral cortex (Murakami and Furuse 2010; Hoffman et al. 2015a, 2017). BDNF plays a key role in brain health by promoting synaptic plasticity and neurogenesis (Wetmore et al. 1990; Figurov et al. 1996). Increases in BDNF may stimulate increased dendritic branching and proliferation of cells within the hippocampus, therefore playing an essential role in cognitive function and memory (Erickson et al. 2010).

Military deployment and SUSOPs may occur with limited lead time. Most studies examining the beneficial effects of BA supplementation on performance range from 4 to 8 weeks in duration (Hoffman et al. 2018), which may not be practical for use in military personnel with short notice before a mission. A recent investigation by Church et al. (2017) demonstrated that a 2 week supplement duration of 12 g/day of BA increased intramuscular carnosine content to the same extent as 6 g/day taken for 4 weeks. In addition, the greater supplement dose was not associated with any greater risk for adverse effects (Church et al. 2017). Therefore, the purpose of this investigation was to examine the effects of 2 weeks of BA supplementation on physical performance, cognitive function, inflammation, and mood during a 24 h simulated SUSOP.

## Materials and Methods

### Experimental design

**Figure 1:**
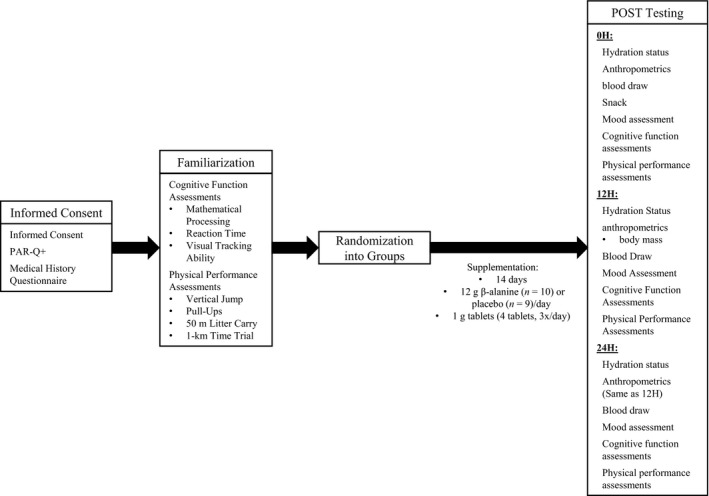
Depiction of all assessments and testing time points. POST: after 14‐days of β‐alanine or placebo supplementation.

Each participant reported to the Human Performance Laboratory on four occasions. On the first occasion, participants completed a medical history questionnaire, and a physical activity readiness questionnaire (PAR‐Q+). On the second occasion, participants experienced a familiarization session with all of the physical and cognitive performance assessments to minimize any learning effect of the assessments on outcome measures. On the third occasion, participants were randomly assigned to one of two groups: sustained‐release BA or PLA. Each participant was instructed to consume 12 g/day of their respective supplement for 14 days, for a total ingestion of 168 g. Following the 14 day supplementation period, participants returned to the laboratory for their final testing visit (POST), which consisted of a 24 h simulated SUSOP. Throughout the 24 h SUSOP, participants endured military‐specific tasks designed to simulate combat situations, including mission briefs, weighted ruck marches, and caloric restriction. During POST testing, all assessments were performed at three separate time points: upon arrival to the laboratory (0H), 12 h after arrival (12H), and 24 h after arrival (24H). A depiction of all assessments and time points is presented in Figure 1.

### Participants

Forty recreationally active males between the ages of 18 and 35 were recruited for this study. This was a double‐blind study, in which participants were assigned to either BA or PLA in a counterbalanced fashion. This investigation was approved by the University of Central Florida Institutional Review Board for human subjects, and all procedures were in accordance with the ethical standards of the 1964 Helsinki Declaration and its later amendments. Following an explanation of all procedures, risks, and benefits, each participant provided their written informed consent to participate in the study. Additionally, all participants were required to be free of any physical limitations (as determined by a medical history questionnaire and PAR‐Q+) and were defined as recreationally active (Riebe et al. 2018). None of the participants recruited were vegetarians. Furthermore, participants did not consume BA for at least 9 weeks prior to enrollment in the study and did not consume creatine for at least 4 weeks prior to enrollment in the study. Although previous military experience was not part of the inclusion criteria for this investigation, six participants had previous military experience, 10 participants were actively involved in the U.S. Army Reserve Officer Training Corps (ROTC) at the University of Central Florida, and 24 participants did not have previous military experience. It is possible that some of the participants recruited may have had previous SUSOP experience, however, it is unlikely that these SUSOPs would have been performed in controlled environments; therefore we believe that the 24 h SUSOP utilized in this investigation was a unique and novel stimulus for all of the participants.

### Supplementation

Each participant was instructed to consume 4 g of their respective supplement three times daily for a total of 12 g/day for 2 weeks for a total of 168 g with food and water. Participants were instructed to consume the supplement with regular meals (breakfast, lunch, dinner). Both the BA and PLA were provided in tablet form (1 g each) and were identical in appearance. The BA was a sustained‐release formulation, and the PLA consisted of rice powder. Supplement compliance was tracked by self‐reported supplement logs and by inspection of the number of pills in each bottle upon return for POST testing. To remain in the final analysis, participant compliance was set at 90% compliance.

### Nutrient intake and dietary analysis

Participants were instructed to maintain their normal dietary intake habits throughout the investigation. Participants were instructed to keep a three day food log during the supplementation period, consisting of 1 weekend day and 2 weekdays to assess their normal dietary habits. Additionally, participants were required to record everything they consumed for the 24 h before POST testing as accurately as possible, including the type, brand, and amount of food consumed. Total energy and macronutrient intakes were analyzed using FoodWorks Dietary Analysis Software, Version 13 (The Nutrition Company, Long Valley, NJ, USA).

### POST testing and SUSOP

Two weeks after the initiation of supplementation, participants returned to the laboratory for POST testing, which consisted of a 24 h SUSOP. The protocol used for POST testing is depicted in Table 1. All participants were required to refrain from exercise as well as from consuming alcohol and caffeine for at least 24 h prior to POST testing and were required to be at least 10 h fasted. Participants were also instructed to stay euhydrated throughout the 10 h fasting period through consumption of water only. Upon arrival to the laboratory, hydration status, anthropometrics, blood measures, mood, cognitive function, and physical performance were assessed. A standardized snack (total energy: 190 kcal; carbohydrates: 19 g; protein: 7 g; fat: 13 g) was provided to all participants after the blood draw and before all mood, cognitive, and performance assessments were completed.

**Table 1:** A sample layout for the 24 h simulated sustained military operation (SUSOP), encompassing all postsupplementation (POST) testing

Time	Event	Details
0800	0H Testing	Hydration, Anthropometrics, Body Composition, Blood Draw, Snack
0900		Cognitive Function and Mood Assessments
1000		Physical Performance Assessments
1100		
1200	Lecture‐Based Training, Mission Briefs, Combat‐Specific Activities	Introduction / Basic Views on Leadership
1300		Introduction to Insurgency
1400		Infrastructure of an Insurgency
1500		Meal Ready‐to‐Eat, Questions
1600		Counter‐Guerilla Operations
1700		Foreign Internal Defense
1800		Fundamentals of Being a Military Advisor
1900		Reconnaissance
2000	12H Testing	Hydration, Body Mass, Blood Draw
2100		Cognitive Function and Mood Assessments
2200		Physical Performance Assessments
2300		
2400		Equipment (Ruck Sack) Fitting
0100	Ruck March	
0200		
0300	Lecture‐Based Training	Reconnaissance Sketching Activity
0400		Snack
0500	Lecture‐Based Training	Debrief Reconnaissance Activity and Report
0600	Ruck March	
0700		
0800	24H Testing	Hydration, Body Mass, Blood Draw
0900		Cognitive Function and Mood Assessments
1000		Physical Performance Assessments
1100		

Following 0H testing, participants began a 24 h simulated SUSOP. The SUSOP conducted was based on the military experience of several investigators on the research team. The 24 h protocol was designed to minimize down time, while providing short breaks for the participants, however, participants were not permitted to sleep during the 24 h period. During the SUSOP, participants engaged in a series of militarily relevant tasks and skills designed to simulate combat situations, including mission briefs and weighted ruck marches while experiencing caloric restriction. The SUSOP included lectures and exercises covering leadership, insurgency, counter‐guerilla operations, foreign internal defense tactics, military advisor roles, reconnaissance, entering and clearing a room, and patrol base security. Lecture‐based training topics were structured into 1 h blocks, consisting of 40 min lectures, 5 min of practical activity, 5 min of callisthenic exercises, and a 10 min break. During the lecture‐based training, participants were also provided with a standardized Meal‐Ready‐to‐Eat (MRE Star, Sarasota, FL, USA) of their choice, which consisted of a main entrée in the form of a stew, cookies, tortillas, candy, dried fruit or nuts, and a drink mix powder (total energy: 1302.9 ± 86.3 kcal; carbohydrates: 187.7 ± 17.5 g; protein: 35.1 ± 6.0 g; fat: 45.5 ± 11.4 g). Food intake was strictly controlled during the SUSOP, however, water intake throughout the SUSOP was permitted ad libitum.

Twelve hours (12H) after 0H testing, participants underwent another bout of assessments that were similar to those done at 0H. 12H testing consisted of hydration status, body mass, a blood draw, mood, cognitive function, and physical performance assessments. After 12H testing, participants completed two separate 2 h weighted ruck marches on a treadmill. Participants were equipped with a 40 lb. (18.2 kg) ruck sack and were instructed to walk on a treadmill at a speed of 4.8 km/h and 1% incline for 2 h. Temperature and relative humidity were recorded at the beginning and end of each ruck march (Mannix Digital Psychrometer SAM 990DW, New York, NY). Average temperature and humidity ranged during each ruck march between 22.3°C and 23.3°C and 49.0–54.7% respectively. Participants received a 3 h break between each ruck march during which they completed additional lecture‐based training and practical exercises. In addition, they were provided with a standardized snack (total energy: 190 kcal; carbohydrates: 19 g; protein: 7 g; fat: 13 g). Ruck march compliance was recorded for each participant. Temperature and humidity were also monitored for all ruck marches. Twenty‐four hours (24H) after 0H, final assessments were conducted that were identical to those completed at 12H.

### Assessments

#### Hydration status

Hydration status was assessed at 0H, 12H, and 24H. Each participant was asked to provide a urine sample in a sterile container. Urine samples were analyzed for hydration status via refractometry by placing a drop of urine on a refractometer (Human Urine Refractometer, MISCO Refractometer, Cleveland, OH, USA) and visually inspecting its osmolarity. Participants were considered euhydrated if urine osmolarity was less than or equal to 1.020. If the participant was not properly hydrated at the time of assessment, they were asked to drink water and provide another urine sample until properly hydrated. Participants could not continue with the assessments until properly hydrated.

#### Anthropometric measurements

After the participant was confirmed to be properly hydrated, a Health‐O‐Meter Professional scale (Patient Weighing Scale, Model 500 KL, Pelstar, Alsip, IL, USA) was used to measure height (±0.1 cm) at 0H and body mass (±0.1 kg) at 0H, 12H, and 24H.

#### Blood draws

After anthropometric procedures were completed, participants were instructed to lay supine on an examination table for a minimum of 15 min. Blood samples were drawn at 0H, 12H, and 24H. The blood draws collected at 0H were drawn from a forearm vein using a 21 gauge, 1¼ inch Vacutainer^®^ blood collection needle. Blood draws obtained at 12H and 24H were completed using a Teflon cannula placed in a superficial vein, which was accomplished using a plastic syringe. The cannula was flushed periodically with an infusion of a nonheparinized isotonic saline solution (B. Braun Medical Inc., Bethlehem, PA, USA). A total of 20 mL of blood was collected at each time‐point in two, 10 mL Vacutainer^®^ tubes: one containing K_2_EDTA, which is an anticoagulant to prevent the blood from clotting, and a serum tube where the blood was allowed to clot. An aliquot of whole blood from the K_2_EDTA tube was immediately utilized to assess hematocrit and hemoglobin. The remaining blood in the EDTA tube was subsequently centrifuged at 4000*g* for 15 min. Blood in the serum tube was allowed to clot for 30 min at room temperature before being centrifuged. The resulting plasma and serum samples were placed into separate 1.8 mL microcentrifuge tubes and frozen at −80° C for later analysis.

#### Biochemical analyses

Hematocrit concentrations were analyzed in duplicate in whole blood using microcapillary and microcentrifugation (CritSpin, Westwood, MA, USA) techniques. Whole blood hemoglobin was analyzed in duplicate using an automated analyzer (HemoCue, Cypress, CA, USA). Plasma volume shifts were calculated using the formula established by Dill and Costill (1974). The coefficient of variation for hematocrit was 0.87% and was 0.65% for hemoglobin.

Serum concentrations of testosterone, cortisol, and myoglobin and plasma concentrations of BDNF, IL‐6 (High Sensitivity), and CRP, were obtained via commercially available enzyme‐linked immunosorbent assay (testosterone, cortisol, myoglobin: Calbiotech, Spring Valley, CA, USA; BDNF, High‐Sensitivity IL‐6, CRP: R&D Systems, Minneapolis, MN, USA) per manufacturer's instructions. All samples for a particular assay were thawed once and analyzed in duplicate by the same technician using a BioTek Eon spectrophotometer (BioTek Instruments, Inc., Winooski, VT, USA). Intra‐assay coefficients of variation for each assay were: 8.15% for testosterone; 9.21% for cortisol; 7.52% for myoglobin; 7.57% for BDNF; 5.50% for IL‐6; 3.36% for CRP. Interplate coefficients of variation for each assay were: 12.77% for testosterone; 4.85% for cortisol; 15.10% for myoglobin; 17.96% for BDNF; 5.92% for IL‐6; 6.03% for CRP.

### Mood assessments

Mood, cognitive function, and physical performance assessments were administered to each participant by the same researcher at each time point. Assessment instructions were provided to the participants at each testing time point.

#### Visual analog scale

Upon completion of the blood draw, participants were asked to quantify their degree of muscle soreness, pain, focus, and fatigue using a 10 cm visual analog scale (VAS) (Bijur et al. 2001). Participants provided their subjective levels of pain, soreness, focus, and fatigue by making a mark on a horizontal line with words anchored at each end of the VAS. Questions were structured as “How much pain do you feel?” with “I do not feel any pain at all” and “I have never been in more pain” serving as the verbal anchor representing the extreme ratings. The distance from the low anchor to the horizontal line that the participant marked was measured in centimeters. The validity and reliability of VAS in assessing fatigue and energy has been previously established (Lee et al. 1991).

### Cognitive function assessments

#### Mathematical processing

A modified version of the original Serial Sevens Test was used to assess cognitive function (Hayman 1942). The Serial Subtraction Test consisted of a 2 min timed test in which participants were provided a sheet of paper with a list of subtraction equations. Participants were required to subtract the number seven from each random computer‐generated four‐digit number to measure how quickly and accurately they can compute simple mathematical problems. Participants were asked to complete as many calculations as possible in the 2 min period. The number of correct answers was recorded. The test was conducted in a quiet room with no distractions.

#### Reaction time

The Dynavision™ D2 Visuomotor Training Device (D2; Dynavision International LLC, West Chester, OH, USA) was used to assess reaction time (RT), as previously described (Wells et al. 2014). The first assessment (Reaction Test) measured the participant's visual and motor RT to a visual stimulus with the dominant hand. The test was initiated when the participant placed and held their dominant hand on an illuminated “home” button. At this point, a single button would light up (visual stimulus) in one of five locations adjacent to the home button on the same horizontal plane. Once the participant recognized the stimulus, they were required to leave the “home” button, strike the stimulus with their dominant hand and return back to the “home” button. Visual RT was measured as the amount of time it took to identify the stimulus and initiate a reaction by leaving the “home” button. Motor RT was measured as the amount of time it took to physically strike the illuminated button following the initial visual reaction and was measured as the amount of time between the hand leaving the “home” button and striking the stimulus. Time was measured to the nearest one hundredth of a second. Participants were instructed to respond to the stimulus as quickly as possible. For each trial, participants were required to respond to 10 stimuli, and the average reaction time was recorded.

The second assessment (Mode A) measured the participant's ability to react to a stimulus as it changed positions on the board. Following a 5 sec visual countdown on the board's LCD screen, an initial stimulus would present on the Dynavision in a random location. The stimulus remained illuminated until it was struck by the participant. The stimulus would then appear at another random location. The participant was instructed to successfully identify and strike as many stimuli as possible within 60 sec with both hands. Participants were advised to utilize their peripheral vision, keep their hands raised as opposed to down by their sides and avoid crossing the hands over the body. In addition, participants were informed that the stimulus could be struck with any part of the hand. The total number of hits were recorded for each participant.

The third assessment (Mode B) was similar to the previous measure in that participants were required to react to a visual stimulus with both hands as it changed positions on the board, however, a cognitive stress was added as participants were asked to verbally recite a randomly generated five‐digit number that was presented on the LCD screen of the apparatus. A different five‐digit number was presented a total of 11 times throughout the 60 sec test and remained illuminated for 0.75 sec each time. Additionally, the visual stimulus remained illuminated for only 1 sec before changing location, requiring the participant to be increasingly reactive in identifying the stimulus. The participant was instructed to successfully identify and strike each stimulus before it changed position, score as many strikes as possible within 60 sec, and successfully recite all five‐digit numbers. As in Mode A, participants were advised to utilize their peripheral vision, keep their hands raised, avoid crossing the hands over the body and use any part of the hand they desired. The number of successful hits and number of misses were recorded for each participant.

#### Visual tracking ability

Visual tracking speed was assessed by the completion of one core session on the Neurotracker multiple object tracking device (CogniSens Athletic, Inc., Montreal, Quebec, Canada) as previously described (Mangine et al. 2014). Briefly, a core session consisted of 20 individual trials used to quantify spatial awareness by determining the participant's threshold speed for effective perception and processing of visual information sources (Faubert and Sidebottom 2012). For each trial, participants were instructed to sit upright on a stool placed in front of a projection screen. Before each trial, a transparent cube containing eight identical yellow balls, measuring 14 cm in diameter, was presented on the screen. Four of these balls were randomly illuminated for 2 sec before returning to their baseline yellow color. Participants were instructed to track these four balls for the duration of the individual trial. During the trial, all eight yellow balls moved simultaneously in different three‐dimensional paths that were only affected by collisions (impact and bounce) with the wall of the cube and the other balls. Following 8 sec of continuous movement the balls were frozen in place and were each assigned a display number, 1 through 8, by the computer. Participants were instructed to identify the four balls that were originally illuminated at the start of the trial. The speed at which the balls moved on the next trial was dependent on the correct identification of the illuminated balls and was adjusted between trials. If the participant correctly selected all four balls, the speed of the balls was increased. If the participant incorrectly identified even one of the balls, the speed of the balls was reduced for the next trial. At the end of the 20 trials, visual tracking speed was determined to be the fastest speed (in centimeters per second) at which the player could correctly identify, with 100% accuracy, all four illuminated balls. For the first trial, the speed in which the balls moved was standardized to be 68 cm/s. Each participant completed one core session on the Neurotracker at each testing point.

### Physical performance assessments

All physical performance assessments were performed after cognitive function assessments. Prior to all performance assessments, participants were required to complete a standardized dynamic warm‐up, including pedaling on cycle ergometer for 5 min at a self‐selected pace, 10 body‐weight squats, 10 body‐weight walking lunges, 10 dynamic walking hamstring stretches, 10 dynamic walking quadriceps stretches, 10 squat jumps, 10 arm circles, and 10 arm swings.

#### Vertical jump power

To quantify vertical jump (VJ) mean power, participants performed five consecutive counter‐movement jumps. During each counter‐movement jump, participants stood with their hands on their waist and were instructed to maximize the height of each jump, while minimizing the contact time with the ground between jumps. During each jump the participant wore a belt connected to a Tendo™ Power Output Unit (Tendo Sports Machines, Trencin, Slovak Republic). The Tendo™ unit consists of a linear position transducer attached to the end of the belt which measured linear displacement and time. Subsequently, the velocity of each jump was calculated and mean power was determined.

#### Pull‐ups

To assess muscular endurance, participants were asked to complete as many pull‐ups as possible in 60 sec or until volitional failure (whichever came first). A successful pull‐up was evaluated according to published guidelines (Hoffman 2006). Verbal encouragement was provided. The total number of pull‐ups completed in 60 sec was recorded.

#### 50 m litter carry

To assess anaerobic performance at each time point, participants were required to perform a simulated litter casualty carry. Participants were instructed to walk a total distance of 50 m as fast as possible while carrying 165 lb. (75 kg) of additional weight. The weight was distributed through a 65 lb. (29.5 kg) ruck sack on the participant's back and one 50 lb. (22.7 kg) dumbbell in each hand. The dumbbell carry was used to simulate a casualty carry on a litter. Prior to testing, the ruck sack was placed on each participant and the fit was adjusted properly. The participant was then instructed to bend down, pick up the two dumbbells that were placed on the floor at the starting line. Participants began walking to a cone placed 10 m away as fast as possible. Once the participant reached the cone, they were instructed to go around the cone and return to the starting cone 2.5 times for a total distance of 50 m. Verbal encouragement was provided. The time for each participant to complete the casualty carry was recorded.

#### 1 km time trial

To assess aerobic performance at each time point, participants completed a 1 km time trial on a motorized treadmill (Woodway 4Front™, Waukesha, WI, USA). Participants began the test standing on the treadmill and a researcher manually increased the speed to 8.0 km/h. Once the treadmill speed reached 8.0 km/h, participants were able to self‐select their speed and pace throughout the duration of the test. Participants were not allowed to see their speed or the time, however, they were able to see the total distance they had traveled. Participants were instructed to complete 1 km as fast as they could. Verbal encouragement was provided. The time for each participant to run 1 km was recorded.

### Statistical analyses

Prior to statistical procedures, all data were assessed for normal distribution, homogeneity of variance, and sphericity. If the assumption of sphericity was violated, a Greenhouse‐Geisser correction was applied. If normality was violated for a dependent variable, a natural log transformation was applied to all data for that variable. To impute missing data, an expectation maximization algorithm was used. A missing compliantly at random test was used to evaluate that all missing data were deemed to be at random (*P *=* *0.999). In order to keep the variable homogeneous and to increase the correlation between items, the imputation was performed by each respective subscale (i.e., same variable along time points). Outliers in the data set were identified and excluded from data analysis when their data exceeded 2.5 times the median absolute deviation (Leys et al. 2013). To analyze group differences in anthropometric measurements (age, body mass, height) and dietary intake, independent samples t‐tests were used. To analyze changes in dependent variables over the 24 h SUSOP, a 2 × 3 (Group × Time) repeated‐measures analysis of variance (ANOVA) was used. In the event of a significant interaction, LSD post‐hoc tests were used for pairwise comparisons. Because the VAS assessments were non‐normally distributed even after transformations were applied, Mann–Whitney U tests were used to analyze group differences at all time points during the 24 h SUSOP, and Friedman tests were used to determine if changes in mood occurred over time. In the case of a significant chi‐square value, Wilcoxon signed ranks test were used to perform pairwise comparisons. For all analyses, a criterion alpha level of *α *≤ 0.05 was used to determine statistical significance, and statistical software (SPSS V.21.0, Chicago, IL, USA) was used. All data are reported as mean ± standard deviation.

## Results

### Participants

A total of 17 participants withdrew from the investigation prior to supplementation due to reasons unrelated to the study. Three participants withdrew from the study during the supplementation period due to time commitments of POST testing. One participant was dropped from the final analysis because they reported to have consumed creatine throughout the supplementation period. None of the participants withdrew from the investigation during the 24 h SUSOP. A total of 19 participants (PLA: *n *=* *9; BA: *n *=* *10) were included in the final analysis. Of the participants that were included in the final analysis, four participants had previous military experience, two participants were actively involved in the U.S. Army Reserve Officer Training Corps (ROTC) at the University of Central Florida, and 13 participants did not have previous military experience. All data for dependent variables exhibited normality except for mathematical processing ability, BDNF, IL‐6, CRP, and myoglobin concentrations and therefore, a log transformation was applied to these variables.

### Anthropometric measurements

The demographics of participants included in the final analysis are included in Table 2. No significant differences in any of the participant demographic characteristics (age, height, body mass) were observed between groups at 0H (*P *>* *0.05).

**Table 2:** Demographics and anthropometrics of participants included in the final data analysis and body mass changes over the 24 h simulated military operation

Group	*N*	Age (years)	Height (m)	Body mass (kg)		
				0H	12H	24H
BA	10	22.4 ± 3.0	1.75 ± 0.03	80.3 ± 10.9	79.5 ± 10.7	78.3 ± 10.5
PLA	9	23.0 ± 3.8	1.74 ± 0.04	83.6 ± 13.0	83.1 ± 13.0	82.0 ± 12.7
Total	19	22.7 ± 3.3	1.75 ± 0.04	81.8 ± 11.7	81.2 ± 11.6^a^	80.1 ± 11.4^a^^,^ b

Values are presented as mean ± standard deviation. BA, β‐alanine; PLA, placebo; 0H, Upon arrival to the laboratory; 12H, 12 h after 0H testing; 24H, 24 h after 0H testing.

a Significantly different (*P *≤* *0.05) from 0H.

b Significantly different (*P *≤* *0.05) from 12H.

During the SUSOP, a significant effect for time was observed for body mass (*F*_1.395,23.708_ = 57.178, *P *<* *0.001). Body mass was significantly reduced at 12H (*P *=* *0.004) and 24H (*P *<* *0.001) compared to 0H. Body mass was also observed to be significantly lower at 24H compared to 12H (*P *<* *0.001). However, no significant group effect (*F*_1,17_ = 0.433, *P *=* *0.519) or group × time interaction (*F*_1.395,23.708_ = 0.382, *P *=* *0.612) was observed.

### Nutrient intake and dietary analysis

Results of average daily dietary intake for the supplementation period and 24 h SUSOP can be observed in Table 3. Dietary analysis revealed no significant differences in daily nutrient intake between BA and PLA during the supplementation period. However, in the 24 h before the SUSOP, fat intake was significantly greater in BA than PLA (*P *=* *0.024). No other group differences in nutrient intake existed in the 24 h period prior to the SUSOP. Additionally, no group differences existed for nutrient intake during the 24 h SUSOP.

**Table 3:** Average daily dietary intake between groups

	Reported average daily intake during 2 week supplementation period				Reported daily intake for 24 h prior to SUSOP				Total intake during 24 h SUSOP			
	Total energy (kcal)	Carbohydrates (g)	Protein (g)	Fat (g)	Total energy (kcal)	Carbohydrates (g)	Protein (g)	Fat (g)	Total energy (kcal)	Carbohydrates (g)	Protein (g)	Fat (g)
BA	2251.8 ± 653.1	396.1 ± 417.9	140.7 ± 55.4	98.7 ± 36.1	2894.4 ± 1341.9	329.3 ± 201.3	139.6 ± 76.7	118.3 ± 50.7	1503.5 ± 79.4	213.2 ± 19.0	40.9 ± 6.1	57.9 ± 12.1
PLA	2164.8 ± 417.0	221.4 ± 60.1	122.9 ± 32.4	90.2 ± 21.4	2005.4 ± 315.1	242.7 ± 140.0	108.1 ± 67.0	66.8 ± 38.5^a^	1481.1 ± 96.9	199.4 ± 13.0	43.3 ± 5.9	59.2 ± 11.1
Total	2368.5 ± 574.5	313.3 ± 311.4	132.3 ± 45.7	94.7 ± 29.6	2473.3 ± 1129.8	288.3 ± 72.1	124.7 ± 72.1	93.9 ± 51.4	1492.9 ± 86.3	206.7 ± 17.5	42.1 ± 6.0	58.5 ± 11.4

Values are presented as mean ± standard deviation. Reported average daily intake during 2 week supplementation period is based off of a 3‐day self‐reported food log (1 weekend day, 2 weekdays). Reported daily intake for 24 h prior to SUSOP is based off of a 24 h self‐reported food log. Total intake during 24 h SUSOP consisted of two standardized snacks (total energy: 190 kcal; carbohydrates: 19 g; protein: 7 g; fat: 13 g) and a meal‐ready‐to‐eat (total energy: 1302.9 ± 86.3 kcal; carbohydrates: 187.7 ± 17.5 g; protein: 35.1 ± 6.0 g; fat: 45.5 ± 11.4 g). BA, β‐alanine; PLA, placebo.

a Significantly different (*P *≤* *0.05) from BA.

### Supplement and assessment compliance

Supplement compliance was >97% for all participants included in the final data analysis. One participant in BA did not fully complete the second ruck march due to an injury (pulled muscle) and could not complete the lower body physical performance assessments at 24H. One participant in PLA did not fully complete the second ruck march due to nausea, however, all of his other assessments at 24H were completed and included in the final analysis. Additionally, one other participant in BA had to decrease the speed of the treadmill on the second ruck march, but all his assessments at 24H were completed and included in the final analysis.

### Mood assessments

Values for subjective levels of pain, soreness, focus, and fatigue are presented in Table 4. During the 24 h SUSOP subjective feelings of pain increased in both BA (*χ* ^2^ = 14.6, *P *=* *0.001) and PLA (*χ* ^2^ = 13.8, *P *=* *0.001). PLA experienced significantly increased feelings of pain at 12H (*z* = −2.4, *P *=* *0.017) and 24H (*z* = −2.7, *P *=* *0.008) compared to 0H, and significantly greater pain at 24H compared to 12H (*z* = −2.5, *P *=* *0.011). Similarly, participants in BA, experienced greater subjective feelings of pain at 12H (*z* = −2.6, *P *=* *0.009) and 24H (*z* = −2.8, *P *=* *0.005) compared to 0H, and greater pain at 24H compared to 12H (*z* = −2.2, *P *=* *0.028). However, no group differences were noted (*P *>* *0.05) at any time point.

**Table 4:** Changes in subjective mood states during the 24 h simulated military operation between groups

	Pain			Soreness			Focus			Fatigue		
	0H	12H	24H	0H	12H	24H	0H	12H	24H	0H	12H	24H
BA	0.14 ± 0.21	0.95 ± 0.74^a^	3.92 ± 2.92^a^^,^ b	1.51 ± 2.23	2.07 ± 2.43	5.27 ± 1.52^a^^,^ b	7.32 ± 1.45	6.39 ± 1.61^a^	3.40 ± 1.79^a^^,^ b	2.33 ± 2.62	3.40 ± 2.10	7.98 ± 1.48^a^^,^ b
PLA	0.29 ± 0.41	1.16 ± 0.80^a^	5.48 ± 2.19^a^^,^ b	0.38 ± 0.43	1.54 ± 1.11^a^	6.10 ± 2.45^a^^,^ b	8.01 ± 1.30	7.37 ± 1.96	3.93 ± 2.31^a^, ^b^	1.20 ± 0.99	3.11 ± 2.03^a^	8.04 ± 1.39^a^^,^ b

Values are presented as mean ± standard deviation. Mood was assessed using a 10 cm visual analog scale, where each participant was asked to make a vertical mark across a 10 cm horizontal line indicating how much pain, soreness, focus, and fatigue they felt at the time. BA, β‐alanine; PLA, placebo; 0H, upon arrival to the laboratory; 12H, 12 h after 0H testing; 24H, 24 h after 0H testing.

a Significantly different (*P *≤* *0.05) from 0H.

b Significantly different (*P *≤* *0.05) from 12H.

Feelings of soreness were elevated for participants consuming both BA (*χ* ^2^ = 11.1, *P *=* *0.004) and PLA (*χ* ^2^ = 14.2, *P *=* *0.001). PLA experienced greater soreness at 12H (*z* = −2.3, *P *=* *0.021) and 24H (*z* = −2.7, *P *=* *0.008) compared to 0H, and soreness at 24H was significantly higher than 12H (*z* = −2.5, *P *=* *0.011). Similarly, participants consuming BA experienced significantly more soreness at 24H compared to 0H (*z* = −2.497, *P *=* *0.013) and 12H (*z* = −2.090, *P *=* *0.037). However, no differences were observed between 0H and 12H (*z* = −1.6, *P *=* *0.109), and no group differences (*P *>* *0.05) were observed at any time point.

Comparisons of subjective feelings of focus revealed significant changes over time in both BA (*χ* ^2^ = 12.8, *P *=* *0.002) and PLA (*χ* ^2^ = 10. 9, *P *=* *0.004). No change in subjective feelings of focus was noted between 0H and 12H (*z* = −0.770, *P *=* *0.441) for PLA. However, focus was significantly reduced at 24H compared to both 0H (*z* = −2.7, *P *=* *0.008) and 12H (*z* = −2.5, *P *=* *0.011). Participants consuming BA were significantly less focused at 12H (*z* = −2.6, *P *=* *0.009) and 24H (*z* = −2.7, *P *=* *0.007) compared to 0H, and at 24H compared to 12H (*z* = −2.5, *P *=* *0.013). Comparisons between groups revealed no significant differences (*P *>* *0.05) at any time point.

Significant changes in subjective feelings of fatigue were noted in both BA (*χ* ^2^ = 16.7, *P *<* *0.001) and PLA (*χ* ^2^ = 14.2, *P *=* *0.001). Fatigue was significantly elevated in PLA from 0H at both 12H (*z* = −2.2, *P *=* *0.028) and 24H (*z* = −2.7, *P *=* *0.008). Fatigue was also greater at 24H compared to 12H (*z* = −2.547, *P *=* *0.011). No change in fatigue was noted in BA between 0H and 12H (*z* = −1.6, *P *=* *0.110), however, subjective feelings of fatigue at 24H were significantly greater than that observed at 0H (*z* = −2.803, *P *=* *0.005) and 12H (*z* = −2.803, *P *=* *0.005). No differences (*P *>* *0.05) in feelings of fatigue were seen between BA and PLA at any time point during the 24 h SUSOP.

### Blood analysis

**Figure 2:**
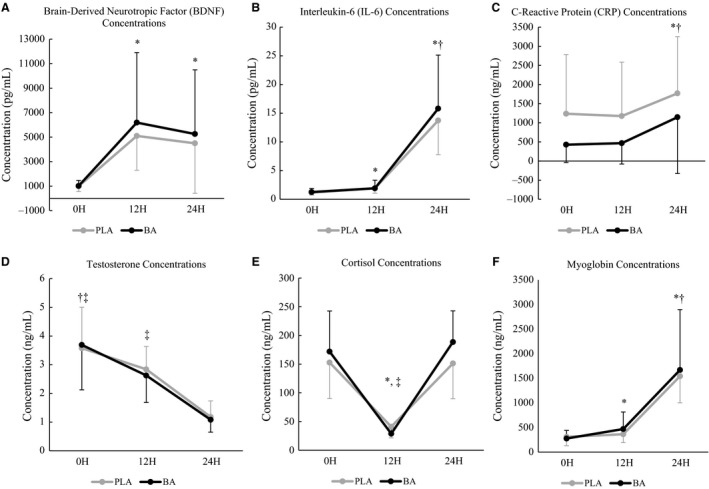
Concentrations of blood analytes (A, brain‐derived neurotropic factor [BDNF]; B, interleukin‐6 [IL‐6]; C, C‐reactive protein [CRP]; D, testosterone; E, cortisol; F, myoglobin) over the 24 h simulated sustained military operation (SUSOP) in participants consuming β‐alanine (BA;* n *=* *10) or placebo (PLA;* n *=* *9) for 14‐days. Means are represented by solid markers, and standard deviations are represented by error bars. 0H: Upon arrival to the laboratory; 12H: 12 h after 0H testing; 24H: 24 h after 0H testing. *Significantly different (*P *≤* *0.05) from 0H when groups are combined. ^†^Significantly different (*P *≤* *0.05) from 12H when groups are combined.

Values for all blood markers measured during the 24 h SUSOP are presented in Figure 2A–F.

#### Brain‐derived neurotropic factor

Changes in circulating BDNF concentrations are depicted in Figure 2A. A significant main effect for time (*F*_1.294,21.995_ = 22.836, *P *<* *0.001) was observed. BDNF concentrations were significantly higher at 12H (*P *<* *0.001) and 24H (*P *<* *0.001) compared to 0H, but no differences were observed between 12H and 24H (*P *=* *0.270). No main effect for group (*F*_1,17_ = 0.063, *P *=* *0.804), nor group × time interaction (*F*_1.294,21.995_ = 0.017, *P *=* *0.940) was noted.

#### Interleukin‐6

Changes in circulating IL‐6 concentrations are depicted in Figure 2B. A significant main effect for time (*F*_2,34_ = 331.996, *P *<* *0.001) was observed. IL‐6 concentrations were significantly higher at 12H (*P *<* *0.001) and 24H (*P *<* *0.001) compared to 0H. IL‐6 concentrations continued to elevate at 24H compared to 12H (*P *<* *0.001). No main effect for group (*F*_1,17_ < 0.001, *P *=* *0.996), nor group × time interactions (*F*_2,34_ = 0.211, *P *=* *0.811) were noted.

#### C‐reactive protein

Changes in circulating CRP concentrations are depicted in Figure 2C. A significant main effect for time (*F*_1.147,19.503_ = 45.675, *P *<* *0.001) was observed. CRP concentrations were significantly elevated at 24H compared to both 0H (*P *<* *0.001) and 12H (*P *<* *0.001), however, no differences were observed between 0H and 12H (*P *=* *0.420). No main effect for group (*F*_1,17_ = 1.529, *P *=* *0.233), nor group × time interactions (*F*_1.147,19.503_ =  0.002, *P *=* *0.981) were noted.

#### Testosterone

Changes in circulating testosterone concentrations are depicted in Figure 2D. A significant main effect for time (*F*_1.187,16.615_ = 33.645, *P *<* *0.001) was observed. Testosterone concentrations were significantly lower at 12H (*P *=* *0.003) and 24H (*P *<* *0.001) compared to 0H. Additionally, testosterone concentrations were significantly greater at 12H compared to 24H (*P *<* *0.001). No main effect for group (*F*_1,14_ = 0.025, *P *=* *0.876), nor group × time interactions (*F*_1.187,16.615_ = 0.178, *P *=*  *0.721) were observed.

#### Cortisol

Changes in circulating cortisol concentrations can be observed in Figure 2E. A significant effect for time (*F*_1.290,18.055_ = 62.055, *P *<* *0.001) was observed. Cortisol concentrations at 12H were significantly lower than that observed at both 0H (*P *<* *0.001) and 24H (*P *<* *0.001). Additionally, cortisol concentrations were significantly greater at 24H compared to 0H (*P *=* *0.041). No main effect for group (*F*_1,14_ = 0.053, *P *=* *0.822), nor group × time interactions (*F*_1.290,18.055_ = 1.279, *P *=* *0.286) were observed.

#### Myoglobin

Changes in circulating myoglobin concentrations can be observed in Figure 2F. A significant main effect for time (*F*_1.351,18.918_ = 94.281, *P *<* *0.001) was noted. Myoglobin concentrations were significantly greater at 12H (*P *<* *0.001) and 24H (*P *<* *0.001) compared to 0H. In addition, myoglobin concentrations were significantly greater at 24H compared to 12H (*P *<* *0.001). No main effect for group (*F*_1,14_ = 0.098, *P *=* *0.759), nor group × time interactions (*F*_1.351,18.918_ = 1.260, *P *=* *0.291) were noted.

#### Plasma volume shifts

No significant main effects for group (*F*_1,16_ = 1.192, *P *=* *0.291), time (*F*_1,16_ = 1.399, *P *=* *0.254, partial *η* ^2^ = 0.080), or group × time interaction (*F*_1,16_ = 0.001, *P *=* *0.977) were observed for plasma volume shifts. When groups were combined, plasma volume shifts were equal to 2.56 ± 3.15% from 0H to 12H and 3.65 ± 4.28% from 0H to 24H. Circulating markers were not corrected for changes in plasma volume due to the importance of molar exposure at the tissue receptor level.

### Cognitive function assessments

Results for cognitive function assessments are presented in Table 5.

**Table 5:** Changes in cognitive function assessments during the 24 h simulated military operation between groups

	Mathematical processing‐number of correct calculations in 2‐min (#)			Dynavision motor reaction time (s)			Dynavision visual reaction time (s)			Dynavision number of hits in 60‐sec (#)		
	0H	12H	24H	0H	12H	24H	0H	12H	24H	0H	12H	24H
BA	23.7 ± 9.5	25.3 ± 8.4	24.4 ± 9.0	0.30 ± 0.06	0.30 ± 0.06	0.31 ± 0.05	0.36 ± 0.03	0.36 ± 0.04	0.35 ± 0.05	85.9 ± 6.2	84.9 ± 5.6	84.5 ± 5.5
PLA	23.4 ± 8.3	26.3 ± 8.8	24.8 ± 6.6	0.26 ± 0.05	0.23 ± 0.03	0.26 ± 0.05	0.35 ± 0.03	0.36 ± 0.04	0.39 ± 0.05^a^	89.1 ± 5.9	89.4 ± 6.6	85.1 ± 3.4
Total	23.6 ± 8.8	25.7 ± 8.4^a^	24.6 ± 7.8	0.28 ± 0.06	0.26 ± 0.05	0.29 ± 0.06	0.36 ± 0.03	0.36 ± 0.04	0.37 ± 0.05	87.3 ± 6.1	86.9 ± 6.3	84.8 ± 4.6

	Dynavision number of hits in 60‐sec with distraction (#)			Dynavision number of misses in 60‐sec with distraction (#)			Neurotracker speed threshold (a.u.)		
	0H	12H	24H	0H	12H	24H	0H	12H	24H
BA	75.7 ± 7.4	77.4 ± 9.4	70.2 ± 9.1	9.2 ± 3.3	7.3 ± 3.9	11.2 ± 1.7	1.57 ± 0.46	1.51 ± 0.26	1.33 ± 0.26
PLA	81.6 ± 5.9	77.6 ± 6.2	70.6 ± 7.8	6.0 ± 3.4	10.6 ± 1.6^a^^,^ c	11.0 ± 1.0^a^	1.64 ± 0.39	1.35 ± 0.36	1.44 ± 0.21
Total	78.3 ± 7.2	77.5 ± 7.9	70.4 ± 8.3^a^^,^ b	7.5 ± 3.6	9.1 ± 3.3	11.1 ± 1.3^a^^,^ b	1.60 ± 0.42	1.43 ± 0.32	1.38 ± 0.23

Values are presented as mean ± standard deviation. BA, β‐alanine; PLA, placebo; 0H, upon arrival to the laboratory; 12H, 12 h after 0H testing; 24H, 24 h after 0H testing.

a Significantly different (*P *≤* *0.05) from 0H.

b Significantly different (*P *≤* *0.05) from 12H.

c Significantly greater (*P *≤* *0.05) than participants consuming the β‐alanine.

#### Mathematical processing

A significant main effect for time (*F*_2,32_ = 6.134, *P *=* *0.006) was observed for the number of correct calculations performed in 2‐min. With groups combined, a significantly greater amount of correct calculations was noted at 12H compared to 0H (*P *=* *0.003). No other significant differences were observed between 0H and 24H (*P *=* *0.083) or between 12H and 24H (*P *=* *0.116). In addition, no significant main effects for group (*F*_1,16_ = 0.028, *P *=* *0.868) or a group × time interaction (*F*_2,32_ = 0.211, *P *=* *0.811) was observed.

#### Reaction time

##### Reaction test

A significant main effect for group (*F*_1,16_ = 7.272, *P *=* *0.016) was observed for motor RT. Participants consuming BA had a significantly slower motor RT compared to PLA. However, no main effect for time (*F*_2,32_ = 1.473, *P *=* *0.244) nor group × time interaction (*F*_2,32_ = 0.294, *P *=* *0.748) was observed. A significant group × time interaction was discovered for visual RT (*F*_2,34_ = 5.788, *P *=* *0.007). *Post hoc* analyses revealed that participants consuming PLA experienced significant changes (*F*_2,16_ = 4.443, *P *=* *0.029) in visual RT over the 24H. Visual RT in PLA was significantly slower at 24H compared to 0H (*P *=* *0.035), but no other significant differences were noted. Conversely, no significant changes in visual RT occurred for participants consuming the BA (*F*_2,18_ = 1.436, *P *=* *0.264). Furthermore, no group differences in visual RT existed at any time point (0H: t = −0.804, *P *=* *0.433, 12H: t = −0.095, *P *=* *0.925, 24H: t = 1.671, *P *=* *0.113). Additionally, no effects for time (*F*_2,34_ = 1.396, *P *=* *0.261) or group (*F*_1,17_ = 0.227, *P *=* *0.640) were observed for visual RT.

##### Mode A

No significant main effects for group (*F*_1,16_ = 1.419, *P *=* *0.251), time (*F*_2,32_ = 3.232, *P *=* *0.053), or group × time interaction (*F*_2,32_ = 1.457, *P *=* *0.248) were observed for the number of successful hits completed in 60‐sec without a distraction.

##### Mode B

A significant main effect for time (*F*_2,28_ = 13.077, *P *<* *0.001) was observed for the number of successful hits completed in 60‐sec with a distraction. Significantly fewer hits were completed at 24H compared to 0H (*P *<* *0.001) and 12H (*P *=* *0.001), but no differences were observed between 0H and 12H (*P *=* *0.572). However, no significant main effects for group (*F*_1,14_ = 0.384, *P *=* *0.545) or group × time interaction (*F*_2,28_ = 1.760, *P *=* *0.190) were observed. In addition to the number of successful hits during this specific assessment, the total number of misses that occurred within the 60‐sec test with a distraction was also recorded. No significant effect for group (*F*_1,11_ = 0.002, *P *=* *0.967) was observed, however, a significant main effect for time (*F*_2,22_ = 4.940, *P *=* *0.017) was seen. The number of misses was significantly greater at 24H compared to 0H (*P *=* *0.009) and 12H (*P *=* *0.023), with no differences noted between 0H and 12H (*P *=* *0.341). In addition, a significant group × time interaction (*F*_2,22_ = 4.077, *P *=* *0.031) was observed. A significant difference existed between groups at 12H (*P *=* *0.048), with BA having significantly less misses than PLA, however, no difference was noted at 0H (*P *=* *0.242) or 24H (*P *=* *0.832). Furthermore, no significant change in the number of misses in BA was noted during the SUSOP (*F*_2,10_ = 1.937, *P *=* *0.195), while a significant change (*F*_2,12_ = 9.904, *P *=* *0.003) in the number of misses during this period was observed in PLA. *Post‐hoc* analyses revealed that the number of misses was significantly greater at 12H (*P *=* *0.032) and 24H (*P *=* *0.004) compared to 0H. No other differences were noted.

#### Visual tracking ability

No significant main effects for time (*F*_2,32_ = 2.827, *P *=* *0.074), group (*F*_1,16_ = 0.003, *P *=* *0.961), or group × time interaction (*F*_2,32_ = 1.197, *P *=* *0.315) were observed.

### Physical performance assessments

Values for physical performance assessments during the 24 h SUSOP are presented in Table 6.

**Table 6:** Changes in physical performance assessments during the 24 h simulated military operation between groups

	Vertical jump mean power (W)			Pull‐ups in 60‐sec (#)			50‐m litter carry time (s)			1‐km time trial time (s)		
	0H	12H	24H	0H	12H	24H	0H	12H	24H	0H	12H	24H
BA	1193.1 ± 164.1	1217.3 ± 164.3	1189.3 ± 163.1	14.8 ± 3.8	14.6 ± 4.1	14.8 ± 3.7	24.79 ± 3.19	23.94 ± 3.36	25.19 ± 4.15	290.0 ± 46.2	297.3 ± 42.9	319.4 ± 42.9
PLA	1212.4 ± 117.2	1195.3 ± 153.2	1154.2 ± 111.6	16.3 ± 5.7	16.8 ± 5.7	16.5 ± 5.4	23.16 ± 2.94	22.91 ± 2.76	25.79 ± 5.44	288.1 ± 22.0	297.5 ± 25.8	357.6 ± 41.9^c^
Total	1201.7 ± 141.5	1207.5 ± 155.2	1173.7 ± 139.7	15.5 ± 4.7	15.6 ± 4.8	15.6 ± 4.5	24.07 ± 3.10	23.48 ± 3.06^a^	25.45 ± 4.63^a^^,^ b	289.2 ± 36.5	297.4 ± 35.3^a^	336.4 ± 46.6^a^^,^ b

Values are presented as mean ± standard deviation. BA, β‐alanine; PLA, placebo; 0H, upon arrival to the laboratory; 12H, 12 h after 0H testing; 24H, 24 h after 0H testing.

a Significantly different (*P *≤* *0.05) from 0H.

b Significantly different (*P *≤* *0.05) from 12H.

c Significantly greater (*P *≤* *0.05) than participants consuming the β‐alanine.

#### VL power

No significant effects for time (*F*_2,32_ = 3.036, *P *=* *0.062), group (*F*_1,16_ = 0.034, *P *=* *0.857), or group × time interaction (*F*_2,32_ = 1.702, *P *=* *0.198) were observed for VJ mean power.

#### Pull‐ups

No significant main effects for time (*F*_1.421,21.311_ = 0.044, *P *=* *0.908), group (*F*_1,15_ = 0.651, *P *=* *0.432), or group × time interaction (*F*_1.421,21.311_ = 0.254, *P *=* *0.702) were discovered for pull‐ups completed during the 24 h SUSOP.

#### 50 m litter carry

A significant main effect for time (*F*_1.190,19.040_ = 9.691, *P *=* *0.004) was observed for 50 m litter carry completion time. Litter carry time at 12H was significantly faster than both 0H (*P *=* *0.015) and 24H (*P *=* *0.002). In addition, litter carry completion time at 24H was significantly slower (*P *=* *0.020) compared to 0H. However, no significant main effect for group (*F*_1,16_ = 0.167, *P *=* *0.688) or group × time interaction (*F*_1.190,19.040_ = 2.806, *P *=* *0.105) was observed.

#### 1 km time trial

No significant main effect for group (*F*_1,16_ = 0.503, *P *=* *0.488) was noted for 1 km time. However, a significant main effect for time (*F*_1.447, 23.155_ = 40.556, *P *<* *0.001) was seen, with post hoc analyses revealing that the time to complete a 1 km run was significantly slower at 12H (*P *=* *0.036) and 24H (*P *<* *0.001) compared to 0H. In addition, time for the 1 km time trial was also significantly slower at 24H compared to 12H (*P *<* *0.001). Furthermore, a significant group × time interaction (*F*_1.447,23.155_ = 7.356, *P *=* *0.007) was observed. Participants consuming BA were significantly faster at 24H (t = 2.095, *P *=* *0.050) compared to PLA. No other between group differences were noted at 0H (t = 0.348, *P *=* *0.732) or 12H (t = 0.012, *P *=* *0.991).

## Discussion

Although the effects of BA supplementation on physical performance have been well‐documented (Hoffman et al. 2018), this is the first study to investigate the effects of BA supplementation on performance during a simulated SUSOP. Similar to previous SUSOP field studies (Nindl et al. 2007; Solis et al. 2015), this laboratory‐based investigation of a 24 h SUSOP resulted in an increased inflammatory response and significant impairments of a number of physical performance, cognitive function, and mood measures. The main findings of this study indicated that daily ingestion of 12 g BA for a period of 14 days resulted in maintenance of reaction time with cognitive stress, cardiovascular endurance, and mood during a simulated 24 h SUSOP, but did not reduce the endocrine and inflammatory response.

Recent investigations have reported that chronic BA supplementation is beneficial for maintaining physical performance in soldiers during intense military training. Hoffman et al. (2014) reported that 4 weeks of BA supplementation (6.0 g/day) was effective in maintaining lower body power, psychomotor performance, peak jump power, target engagement speed, and shooting accuracy in soldiers during intense military training. A subsequent study using the same dosing protocol (6.0 g/day for 4‐weeks) resulted in a significantly faster time for a 50 m casualty carry in soldiers supplementing with BA compared to soldiers consuming the PLA (Hoffman et al. 2015a). This present investigation appears to be the first study to examine the effects of BA supplementation during a 24 h SUSOP consisting of sleep deprivation, caloric restriction, and physical exertion.

Caloric intake was limited to 1493 kcal throughout the 24 h SUSOP, which consisted of one MRE (1100–1300 kcal), and two standardized snacks (190 kcal each). This was comparable to the daily nutrient consumption during typical military training (Nindl et al. 2002; Tharion et al. 2005; Lieberman et al. 2006). Participants lost a significant amount of body mass over the 24 h period (2.07%; 1.7 kg), but no differences were noted between BA and PLA. This weight loss is consistent with previous studies reporting significant body mass loss during SUSOPs (Nindl et al. 2002; Lieberman et al. 2006). The body mass loss in this investigation does not appear to be related to dehydration as participants were allowed to drink water ad libitum and were euhydrated at all assessments. Although speculative, decreases in body mass during the 24 h SUSOP are likely attributed to caloric restriction and muscle protein loss, as significant decreases in muscle mass have been observed with short‐duration military operations (Nindl et al. 2002; Tipton et al. 2003).

The SUSOP resulted in impairments in physical performance, which were similar to those previously reported in SUSOPs of longer duration (Legg and Patton 1987; Knapik et al. 1990; Nindl et al. 2002). In the current study, significant increases were observed in the time to complete the 50 m simulated litter carry and 1 km run at 24H. Previous research has shown that VO_2_max declines with underfeeding (Montain and Young 2003) and sustained military exercise (Guezennec et al. 1994). The decline in aerobic capacity is still apparent during a SUSOP even when caloric balance is maintained (Jacobs et al. 1989), which may be attributed to the accumulated fatigue resulting from the prolonged, repetitive physical tasks (Nindl et al. 2002). Participants in the present study likely experienced accumulated fatigue from a combination of unfamiliarity of the SUSOP, the testing protocol, prolonged wakefulness, and the ruck marches. Although speculative, the repetitive physical demands of the ruck marches alone may have reduced lower‐body aerobic endurance, affecting 1 km run and 50 m litter carry performance. Participants were required to complete two, 2 h ruck marches between 12H and 24H. Improvements in 50 m litter carry time at 12H, but slower times at 24H are suggestive of an accumulated fatigue occurring from the multiple ruck sack marches in addition to prolonged wakefulness and physical exertion. Although the time to complete the 50 m litter carry was not significantly different between BA and PLA, 1 km time was significantly lower in BA compared to PLA after 24H. These findings are consistent with previous research indicating that chronic BA supplementation is most effective for improving high‐intensity exercise performance lasting between 0.5 and 10‐min (Saunders et al. 2017). The average time of completion of the 50 m litter carry was under 30‐sec, whereas the average time of completion of the 1 km run was ~5‐min.

No differences in VJ power output or pull‐ups completed in 60 sec were noted in either group. These findings are consistent with those of Knapik et al. (1990) who reported no changes in lower‐body power performance after a 5 day military operation. However, these findings contrast with those of Hackney et al. (1991) and Nindl et al. (2002), who reported significant decreases in lower‐body anaerobic power after 4.5 days and 72 h of SUSOPs, respectively. Additionally, Knapik et al. (1990), observed significant reductions in upper‐body strength in the 5 day SUSOP. These latter studies were of far greater duration than the present study. In addition, as discussed previously, the ruck marches would have the greatest fatiguing effect on lower body performance, which can explain why no changes were noted in the present study for pull‐up performance. Furthermore, maintenance of VJ power in both groups during the 24 h SUSOP likely reflect a limited neuromuscular stress during the 24 h SUSOP, as compared to the likely greater metabolic stress resulting from prolonged activity, food restriction, and sleep deprivation. An important note to make is that most participants in the current study were not able to continue completing pull‐ups after the 30 sec mark, and many individuals dropped off of the bar before the 60 sec period was over. This is not surprising, as one of the main physiological roles of carnosine is to act as a buffer of H^+^ (Hobson et al. 2012). Exercises requiring maximal strength and power or muscular endurance likely do not stress the anaerobic system enough to elicit a significant elevation in H^+^ concentrations. Furthermore, most studies have reported no significant increases in maximal strength and power or muscular endurance (lasting less than 60 sec in duration) after BA supplementation (Hannah et al. 1985; Hoffman et al. 2008b; Kendrick et al. 2008; Jones et al. 2017).

Changes in testosterone and cortisol concentrations provide an indicator of the anabolic and catabolic status of the body, respectively, and these hormones have previously been used to quantify stress associated with prolonged military activity (Lieberman et al. 2005a,2005b; Nindl et al. 2007). Previous investigations have reported significant elevations in cortisol concentrations and significant decreases in testosterone concentrations during military training (Friedl et al. 1985; Opstad 1992; Lieberman et al. 2005a; Nindl et al. 2007). Increases in cortisol concentrations have been shown to be associated with performance decrements (Lieberman et al. 2005a). In the present study, testosterone concentrations were significantly decreased after 12H and continued to decrease at 24H, while cortisol concentrations were decreased at 12H, but were significantly elevated at 24H. The decrease in the testosterone/cortisol ratio at 24H was indicative of a catabolic state, which may have contributed to the decreases in physical performance. An important point of consideration is that cortisol release exhibits a circadian rhythmicity, with concentrations peaking in the morning and dropping at night (Krieger et al. 1971; Weitzman et al. 1971; Debono et al. 2009). However, in the present study, the increase in cortisol concentrations at 24H compared to 0H indicates a greater amount of stress placed on the body at 24H compared to 0H. Thus, a decreased in the testosterone/cortisol ratio at 24H indicates a catabolic state, which may underlie decreases in physical performance. The lack of any group differences in testosterone or cortisol concentrations during the 24 h SUSOP suggested that BA supplementation was not effective in maintaining the anabolic/catabolic response, which is consistent with previous research (Hoffman et al. 2008a).

Increases in inflammatory (IL‐6 and CRP) and muscle damage (myoglobin) markers have been shown to increase during SUSOPs with sleep deprivation or sleep restriction (Gomez‐Merino et al. 2003; McClung et al. 2013). The results of this present study were consistent with these findings. The greatest increase in these markers were seen between 12H and 24H, which corresponded to the most physically demanding portion of the SUSOP. Similar to the endocrine response, there were no group differences in IL‐6, CRP, or myoglobin concentrations. These results were not consistent with recent animal studies suggesting that when BA ingestion occurs prior to stressful stimuli, elevations in brain carnosine content is associated with an increased resiliency to various stressors by reducing the inflammatory response (Hoffman et al. 2015a, 2017). Whether elevations in muscle carnosine content has similar anti‐inflammatory or muscle protective properties is not well‐understood. Further, protective effects within the brain observed from elevated carnosine has not been observed in humans, and whether elevated inflammatory markers seen in the circulation reflect the inflammatory milieu within muscle, brain or the combination of two requires further study.

Elevations in carnosine content within the brain may serve as a neural protectant through its action as an antioxidant (Kohen et al. 1988), and previous research has demonstrated that chronic BA supplementation can increase brain carnosine concentrations in murine models (Murakami and Furuse 2010; Hoffman et al. 2015b). These elevations also appear to increase the expression of BDNF (Murakami and Furuse 2010; Hoffman et al. 2015a, 2017), which may promote synaptic plasticity and neurogenesis (Wetmore et al. 1990; Figurov et al. 1996), therefore improving cognitive function and memory (Erickson et al. 2010). In the present study, BDNF concentrations were elevated from 0H to 12H, and continued to stay elevated at 24H. Previous research has shown that chronic periods of stress, sleep disturbance, and military training lead to a downregulation of BDNF expression (Suzuki et al. 2014), however acute periods of stress (e.g., sleep deprivation, caloric deficit, and physical exertion) may increase the BDNF response (Gronli et al. 2013; Huang et al. 2014; Schmitt et al. 2016). BDNF plays an essential role in cognitive function and memory (Erickson et al. 2010), and transient increases in BDNF may explain the maintenance of some aspects of cognitive function during the SUSOP. However, there were no group differences in BDNF concentrations at any of the time points, contradicting the findings of other investigations (Murakami and Furuse 2010; Hoffman et al. 2015b). Nevertheless, these previous studies used a rat model. Although BA supplementation in murine models have been shown to elevate brain carnosine levels, this has yet to be demonstrated in any human study.

Physical stress seems to be the primary factor underlying decreased physical performance rather than cognitive function in a SUSOP (Nindl et al. 2002), whereas sleep deprivation appears to have a greater effect on cognitive function than on physical performance (Horne et al. 1983; Haslam 1984). During periods of prolonged operational stress, impairments in cognitive function have been reported, including reduced reaction time and target accuracy, poor reasoning and memory, lack of concentration, poor judgment, and hallucinations (Vrijkotte et al. 2016). Several investigations have indicated that cognitive function, executive performance and mood may be more sensitive to a SUSOP than physical performance (Haslam 1984; Lieberman et al. 2006). In the present study, significant increases in pain, soreness, and fatigue, and a decrease in focus were observed, which was consistent with previous studies (Lieberman et al. 2002, 2006; Nindl et al. 2002). Cognitive measures including reaction assessments and visual tracking ability were maintained during the 24 h SUSOP, however, when a distraction was added to the reaction assessments, significantly more errors were made. The process of trying to focus on many variables at once and make decisions requires extensive amounts of reasoning and higher‐level cognitive functioning, which requires a longer recovery time (Vrijkotte et al. 2016), and may explain the reduction in performance on these measures. In contrast, mathematical processing ability, motor RT, visual RT, and visual tracking ability do not require a high level of reasoning and may not be impacted by a 24 h SUSOP.

The maintenance of cognitive functioning abilities, and in particular, higher‐level brain processes during a SUSOP is crucial to mission success and minimizing mistakes in executive functioning. The results of this present study indicated that BA supplementation may have provided potential cognitive benefits. Participants consuming the PLA had significantly slower visual RT at 24H compared to at 0H, whereas participants consuming the BA maintained their visual RT over time. In addition, higher‐level cognitive functioning was elevated in participants consuming the BA, as participants consuming the PLA made significantly more mistakes during RT testing with a distraction at 12H compared to BA, which was significantly elevated compared to 0H and continued to stay elevated after 24H. These findings were similar to previous studies who reported an increase in the number of correct responses on the Serial Sevens Test in the shooting range while continuous live fire was being directed at targets (Hoffman et al. 2015a), and improved marksmanship while overcoming a misfire and target engagement speed while fatigued (Hoffman et al. 2014). These results suggest that BA supplementation may be beneficial for improving cognitive function on tasks performed under stressful situations, rather than on tasks that are already well‐learned.

In addition to the beneficial effects of BA on cognitive function, several investigators, examining various stress models in rodents have reported that BA supplementation can reduce anxiety and depression (Tomonaga et al. 2008; Murakami and Furuse 2010; Hoffman et al. 2017). These studies linked the anxiolytic effects of BA supplementation on elevations in brain carnosine and an increased expression of BDNF. Although brain carnosine was not measured in this study, no change in circulating concentrations of BDNF was noted in either group. Whether this reflects an inability to elevate brain carnosine levels from BA supplementation in humans is not well‐understood. However, mood patterns did appear to differ between BA and PLA. Feelings of soreness and fatigue were observed in PLA at 12H, whereas increases in subjective feelings of soreness and fatigue were not seen until 24H in participants consuming BA. Subjective feelings of focus were significantly decreased after 12H in BA, but not in PLA. This latter effect is difficult to explain and may be spurious in nature.

There are several limitations to the present study that may have impacted the results. Many of the participants did not have previous military experience, and extrapolation to an actual military population may be difficult. Previous research has suggested that soldiers with SUSOP experience have lower anxiety and better cognitive function than those with less experience (Lieberman et al. 2005a). In addition, the duration of the SUSOP utilized in this investigation was 24 h, whereas most studies examining the effects of SUSOPs last multiple days without complete sleep deprivation. However, the decline in physical performance, cognitive function, and mood observed in this study was comparable to those found in longer‐duration SUSOP studies. Another potential limitation to the study was that muscle and brain carnosine content was not measured, so it is left to speculation that group differences in outcome variables are related to increases in carnosine concentrations. In addition, the dropout rate of participants was high, leading to a smaller than desired sample size. In conclusion, this investigation demonstrated that 12 g/day of BA supplementation for 14 days resulted in several improvements in physical performance, cognitive function, and mood during a 24 h simulated SUSOP. However, BA supplementation did not appear to influence markers of inflammation, muscle damage, anabolic/catabolic status, or neurotrophin concentrations.

## Disclosures

The authors have no conflict of interests to disclose.
